# Effects of Landscape Context on Bird Community in the Subtropical Evergreen Broad-Leaved Forest of Wuyishan National Park

**DOI:** 10.3390/ani13081294

**Published:** 2023-04-10

**Authors:** Yi Wu, Wenwen Zhang, Yifei Wang, Shengjun Zhao, Jing Tian, Jie Shi, Xiao Yang, Peng Cui

**Affiliations:** 1 Research Center for Biodiversity Conservation and Biosafety, State Environmental Protection Key Laboratory on Biosafety, State Environmental Protection Scientific Observation and Research Station for Ecological Environment of Wuyi Mountains, Nanjing Institute of Environmental Sciences, Ministry of Ecology and Environment, Nanjing 210042, China; 2 School of Ecology and Nature Conservation, Beijing Forestry University, Beijing 100083, China

**Keywords:** bird diversity, landscape context, altitude gradient, subtropical evergreen broad-leaved forest, Wuyishan National Park

## Abstract

**Simple Summary:**

Landscape context plays an important role in bird species occurrences and abundance. For local biodiversity conservation and restoration, we examined the effects of landscape context on bird communities at different altitude gradients. The result showed: that (1) species richness and abundance at <300 m altitude were highest among the four altitude gradients, and they showed more significant differences than those at other altitude gradients; (2) the species richness of birds was associated with altitude, season and landscape context, as the season is more significance than other explanatory variables; (3) at the landscape level, habitat configuration is more important. The average canopy height and contagion index positively correlated with the species richness and abundance of birds at all four altitude gradients. In particular, the average canopy height is significant at 300–599 m and 600–899 m altitude gradients. The study results can provide a theoretical basis and guidance for future national park conservation and management and ecological restoration in the subtropical evergreen broad-leaved forest regions.

**Abstract:**

Landscape context can reflect the habitat structure and play an important role in bird species occurrences and abundance. For local biodiversity conservation and restoration, we examined the effects of landscape context on bird communities at different altitude gradients. Our study was conducted in four altitude gradients (<300 m, 300–599 m, 600–899 m, 900–1200 m) of subtropical evergreen broad-leaved forest in Wuyishan National Park, China. The bird survey was carried out in 115 transects in spring, summer, autumn and winter. We examined the effects of altitude, season and landscape context. The result showed that (1) species richness and abundance at <300 m altitude were highest among the four altitude gradients, and they showed more significant differences than those at other altitude gradients; (2) the species richness of birds was associated with altitude, season and landscape context, as the season is more significant than other explanatory variables; (3) at the landscape level, habitat configuration is more important. The average canopy height and contagion index positively correlated with the species richness and abundance of birds at all four altitude gradients. In particular, the average canopy height is significant at 300–599 m and 600–899 m altitude gradients. The study results can provide a theoretical basis and guidance for future national park conservation and management and ecological restoration in the subtropical evergreen broad-leaved forest regions.

## 1. Introduction

Habitat structure is one of the main factors affecting the classification of biological communities [1]. Landscape context can reflect the habitat structure and play an important role in bird species occurrences and abundance [2,3]. However, the spatial extent of local landscape classification decides the landscape context [4]. The number and type of habitats adjacent to a focal habitat are generally defined as the landscape context [5,6]. Additionally, its patch characteristics are also used to analyze the effect on species community [7,8,9].

The impact of landscape context on species has been examined with birds in many regions [10], with examples including forests [11] and farmland [12]. Different responses of forest birds to different forest conditions have been observed in forest landscapes [13,14,15]. The richness and abundance of species are largely dependent on the local forest structures [16,17]. The number of habitats in the landscape will also affect the species composition [18,19]. Meanwhile, the patch characteristics of specific habitat can influence the distribution of species [20]. The landscape context may influence the richness and abundance of specialist and generalist species by altering the species composition [21]. The evidence showed that landscape composition has a greater impact than vegetation structure on forest birds in the artificial forest [22]. Therefore, the numerical response of forest birds to habitat structure can be used to study how landscape drives species richness, richness and diversity [3].

Land cover data can reflect climate change and vegetation change more quickly and effectively [23,24], and they can be used to monitor and assess the wildlife [25]. At the landscape scale, land cover data are widely used to extract characteristics of the landscape context for assessing species diversity. The results of previous studies have shown that changes in landscape configuration (including the amount and proportion of different land cover types, spatial layout, etc.) have differential impacts on the composition of species communities supported by the landscape [26,27,28,29,30]. For example, forest birds are strongly influenced by tree cover, while other bird species are more influenced by landscape-related features [31]. Area, shape, vegetation type and position in the landscape attributes also determine bird community composition, with vegetation type being the most important factor [32]. Landscape fragmentation also affects biological variables (i.e., abundance, richness and probability of occurrence), but there are positive and negative effects [33,34,35]. Studies of Dupont’s larks (*Chersophilus duponti*) show that at the landscape scale, species occurrence is primarily determined by patch size, geographic isolation and interactions between landscape substrates [36]. Isolation was considered the primary cause, followed by landscape matrix composition and patch size, while species density was negatively correlated with patch size. Likewise, patch connectivity and tree height positively influenced bird species richness [37]. Therefore, many landscape attributes influence bird communities. It is necessary to fully understand the interactions between bird communities and different landscape attributes.

Significantly, species responses to landscape context were needed to consider across altitude gradients. Firstly, altitude is an important abiotic environmental factor affecting the distribution of birds [38]. Previous studies have shown that the vertical distribution pattern of species richness has different types in different regions and climatic environmental conditions [39]. A monotonically decreasing pattern is more common in tropical and subtropical regions; i.e., species richness decreases monotonically along an altitudinal gradient, with the highest species richness at lower altitudes. Examples include birds on the Malagasy Islands [40] and reptiles in tropical and subtropical regions [41]. Just as changes in temperature and precipitation caused by climate change will lead to changes in species distribution [42], differences in vegetation productivity, temperature, precipitation, evapotranspiration and other energetic factors caused by altitude differences influence bird abundance [39,43]. Secondly, altitude and landscape usually both affect the distribution of birds [44]. Previous studies have shown that the influence of landscape change on bird community structure and functional diversity depends on elevation [45]. For these reasons, the effects of landscape context on biodiversity should be compared across altitude gradients.

Subtropical evergreen broad-leaved forests are one of the most important vegetation types in southeastern China and are extremely rich in biodiversity. We used multivariate statistics to analyze the relationship between bird diversity and landscape context in broad-leaf evergreen forests at different altitude gradients. This study’s aims were (1) to clarify bird diversity at different altitude gradients, (2) to examine the relationship between landscape context and bird diversity and (3) to identify key landscape factors that influence bird diversity at different altitude gradients. This contributes to local biodiversity conservation and restoration. Earlier studies found that birds are influenced by the spatial distribution of their habitats [46]. Additionally, the communities of forest birds are impacted by seasonal changes in the landscape [9]. Therefore, our study should consider both seasonal variation and spatial scale.

## 2. Materials and Methods

### 2.1. Study Area

Wuyishan National Park (117°24′13″–117°59′19″ E and 27°31′20″–27°55′49″ N), with a total area of 1001.41 km^2^, is located north of the Wuyi Mountains, Fujian Province, China [47,48]. The National Park is a subtropical monsoon climate, and the average annual temperature is 17–19 °C. The range of altitude is wide, with the highest altitude of 2160.8 m and the lowest elevation of 176.1 m. The average annual precipitation is 1684–1780 mm, with the annual relative humidity over 85%. Wuyishan National Park, with forest cover over 93%, is a key area for global biodiversity protection [49]. It covers all vegetation types of subtropical regions and developed the best vertical band spectrum in southeast China [50]. The region is a representative and relatively well-preserved and complete subtropical broadleaf evergreen forest in the subtropical region of China. The vegetation in the region is rich in species and complex in vertical distribution. The vertical band spectrum of vegetation is obvious on the altitude gradient. With the increase in elevation, the vegetation types from bottom to top are broadleaf evergreen forest, mixed coniferous forest, coniferous forest, dwarf forest and subalpine meadow. Wuyishan National Park has complex landforms and diverse ecological environment types, providing an ideal place for wildlife to inhabit and reproduce. According to the statistics of Wuyishan National Park Service, 302 species of birds belonging to 167 genera, 59 families and 18 orders were recorded in national park.

### 2.2. Bird Survey

**Figure 1:**
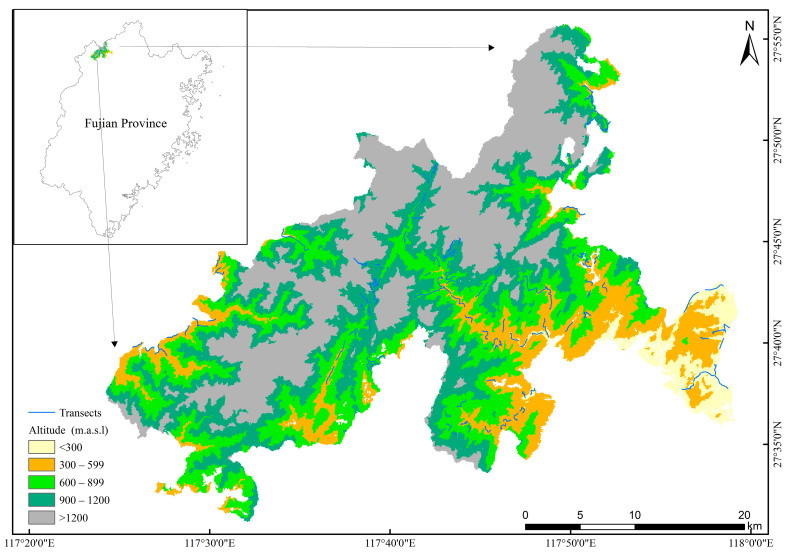
The location of transects in Wuyishan National Park. The coordinate system is CGCS2000_3_Degree_GK_Zone_39.

According to the altitude and vegetation characteristics of the national park, we set the transect below 1200 m where the forest is mainly evergreen broad-leaved forests. A total of 115 transects were set in the study area, and each line was about 1–2 km long (Figure 1). Bird surveys were conducted by the line-transect method [51] during spring (April 2021), summer (July 2021), autumn (October 2021) and wintering (December 2021). The transect was walked along at a speed of 1–2 km/h, and all the birds seen or heard within 50 m on either side were counted. “A field guide to the birds of China [52]” was used to identify bird species.

### 2.3. Field Selection and Environmental Variables

The main objective of this study was to examine the relative role of landscape context in shaping bird communities across different altitude gradients. Transects were divided on the basis of their altitude into four altitude gradients (<300 m, 300–599 m, 600–899 m, 900–1200 m) (Figure 1). Digital elevation data were downloaded from the International Scientific Data Mirror Website of Computer Network Information Center of Chinese Academy of Sciences (http://www.gscloud.cn/, accessed on 13 May 2022). The spatial resolution is 90 m. The number of transects in each altitude gradient were 17, 51, 31 and 16, respectively. To test the impact of the landscape context on a spatial scale, linear buffers with 500 m, 750 m and 1000 m distances were set to form each transect [8,53]. Several studies have demonstrated the relevance of these buffer distances to birds, especially with small forest birds [54,55,56].

The landscape context indices were obtained from the land cover map of Wuyishan National Park and the Chinese forest canopy height dataset. Specific land cover data and forest canopy data were extracted based on the buffer zone extent of each transect. The vector data of Wuyishan National Park’s land-cover was provided by Wuyishan National Park Service, separately for the arbor forest, shrub forest, bamboo, water, construction land, bare land and others. The coordinate system is CGCS2000_3_Degree_GK_Zone_39. We calculated the number of land cover types (N_Type) in each buffer area and selected the percentage of the arbor forest (P_Arbor), percentage of shrub forest (P_Shrub) and percentage of bamboo (P_Bamboo) to represent the landscape area; edge density index (ED) to represent the fragmentation characteristics of the landscape; landscape shape index (LSI) to represent the shape characteristics of the landscape; Euclidean nearest-neighbor distance index (ENN) to represent isolation characteristics of the landscape; Shannon–Wiener landscape diversity index (SHDI) to represent landscape diversity characteristics; contagion index (CONTAG) to represent landscape convergence and connectivity. These indexes of each buffer area were calculated by Fragstats v4.2 (Table 1). ED describes the edge length between patches of heterogeneous landscape elements. The higher edge density index means a higher degree of plaque fragmentation. LSI can characterize the degree of disturbance to the landscape. Generally, the higher value of LSI indicates that the more complex geometric shape of the patch and smaller interference, as the lower value indicates a simpler geometric shape and more interference. ENN describes the distance between patches of the same type. A smaller value of ENN indicates that the closer adjacent distance, the lower degree of isolation, and the better connectivity. SHEI describes the uneven distribution of various patch types in the landscape. When the value of SHEI is small, reflecting that the landscape is dominated by one or a few dominant patch types, the value of SHEI approaches 1, indicating that there are no obvious dominant types in the landscape and that each patch type is evenly distributed in the landscape. CONTAG describes the degree of aggregation or extension trend of different patch types in the landscape. Generally, a high value of CONTAG indicates that a dominant patch type in the landscape with good connectivity. As tall trees may influence the bird species [37], we used the canopy height of China’s forests by integrating GEDI and ICESat-2 data [57]. We extract the average canopy height (A_Height) in each buffer area by ArcGIS 10.6 (Table 1).

### 2.4. Data Analysis

#### 2.4.1. Bird Biodiversity

Shannon–Wiener diversity index (H′), Pielou evenness index (J) and Margalef abundance index (D’) are calculated as follows:

$$H^{\prime }=-\sum_{i}^{S}P_{i}lnP_{i}$$

$$J=H^{\prime }\cdot \ln S^{-1}$$

$$D^{\prime }=(S-1)\cdot \ln N^{-1}$$

where P_i_ is the proportion of the number of individuals of bird species i to the total number of individuals of each transect; S is the number of species recorded in each transect; and N is the total number of individuals observed.

#### 2.4.2. Statistical Analyses

We used species accumulation curves with four altitude gradients to determine whether the sample survey was adequate. One-way analysis [58] of variance was conducted to test for differences in the bird species richness, abundance, Shannon–Wiener diversity index (H′), Pielou evenness index (J) and Margalef abundance index (D′) among four altitude gradients.

We used generalized linear models (GLM) to perform multiple linear regression fitting with full variables. Then, we performed variable screening based on AIC value (Akaike information criterion) step regression to select the fewest variables that could support the model. We also tested the dispersion of the models. In the GLM, the species richness, abundance and Margalef abundance index (D′) in each transect were used as response variables, while the Shannon–Wiener diversity index (H′) and Pielou evenness index (J) were no significance among the four altitude gradients. We used functions GLM and stepAIC from the R package lme4, MASS and DHARMa.

Then, using the variables chosen by step regression, we established spatial linear models (SLM) to prevent the inflation of type I errors and inaccurate parameter estimates caused by spatial auto-correlation. For each model, we examined a range of potential lag distances (1, 2 and 3 km). We used Moran’s I coefficient to assess the level of spatial auto-correlation in the residuals of each model. According to the minimal value of AIC, SLM with a 2 km lag distance was best for both species richness and abundance. The variables with a high z value for their significance in SLM were considered more important influencing factors for species richness and abundance [59,60]. We used function SLM and Moran’s I from the R package spatialreg and spdep.

Finally, we used the constrained ranking method to analyze the relationship between bird species data and environmental factors at different elevation gradients. Both bird species data and environmental factors are multivariate data, and exploratory analysis of the number of individuals of bird variables using detrended correspondence analysis (DCA) is required to determine whether linear or single-peaked ranking methods can be used for analysis. The maximum DCA gradient length for the number of individuals of bird species data was less than 3, so the redundancy analysis (RDA) was selected. The redundancy analysis is a ranking method developed based on correspondence analysis, which combines correspondence analysis with multiple regression analysis and enables analysis of the relationship between two environmental variables and species data. Monte Carlo tests were used to assess model significance, and only models with *p* < 0.05 were shown. The adjusted r^2^ in the RDA model was used to explain the contribution of the selected variables to the variance. The variance obtained by multiplying the adjusted r^2^ by the RDA ranking axes was used as the variance explained for each ranking axis. The environment variables select the landscape variables with high z value both in richness, abundance and Margalef abundance index best SLM. We used function RDA from the R package vegan and ggplot2.

## 3. Results

### 3.1. Bird Community Composition

With statistics, the birds’ data used functions described and aggregated from the R package psych. A total of 16,200 birds of 180 species were recorded in our study, belonging to 17 orders, 55 families and 128 genera. Passeriformes is the largest order among them, with 36 families and 127 species, accounting for 65.45% of the total families and 70.56% of the total species. There are 125 resident species, 44 species of migratory birds and 11 species of passengers. There are two species under first-class state protection, the Yellow-bellied Tragopan *(Tragopan caboti*) and Elliot’s Pheasant (*Syrmaticus ellioti*), and there are 26 species under second-class state protection, accounting for 14.44% of the total. According to the Vertebrate Volume of the Red List of Biodiversity in China, one species of endangered (EN) species was recorded, the *Tragopan caboti*; There are four vulnerable (VU) species, including *Syrmaticus ellioti*, White-necklaced Partridge (*Arborophila gingica)*, Black Eagle (*Ictinaetus malaiensis*) and BlytH′s Kingfisher (*Alcedo herculess*). The number of bird species investigated in spring, summer, autumn and winter were 116, 101, 116 and 120, and the numbers of individuals were 3400, 2264, 4375 and 6044, respectively. Wuyishan National Park has the largest number of birds in winter and the smallest number in summer.

### 3.2. Bird Diversity at Different Altitude Gradient

**Figure 2:**
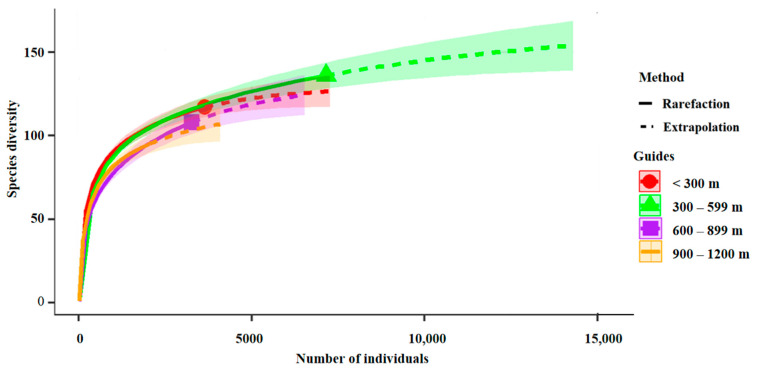
Sample completeness curves for bird communities in four altitude gradients.

The bird population increased sharply at the beginning, and the species accumulation curve leveled off and became saturated when the number of species reached 95 (Figure 2). This indicates that field sampling was adequate for each altitude gradient in Wuyishan National Park.

The bird diversity data in different altitude gradients show that the highest species richness of bird was at <300 m altitude gradient, as the mean value was 20.54; the lowest species richness was at 600–899 m altitude gradient, as the mean value was 17.2 (Table 2). The highest species abundance was at <300 m altitude gradient, as the mean value was 153.01; the lowest species abundance was at 600–899 m altitude gradient, as the mean value was 126.35. The highest Shannon–Wiener diversity index was at 600–899 m altitude gradient, as the mean value was 1.455; the lowest Shannon–Wiener diversity index was at <300 m altitude gradient, as the mean value was 1.298. The highest Pielou evenness index was at 900–1200 m altitude gradient, as the mean value was 0.962; the lowest Pielou evenness index was at <300 m altitude gradient, as the mean value was 0.726. The highest Margalef abundance index was at <300 m altitude gradient, as the mean value was 1.136; the lowest Margalef abundance index was at 600–899 m altitude gradient, as the mean value was 0.758. The mean values of richness, abundance and Margalef abundance index were highest at the <300 m altitude gradient and lowest at the 600–899 m altitude gradient. The species richness and abundance of birds in the evergreen broad-leaved forest of Wuyishan National Park present a gradual decline pattern with the increase of altitude, which conforms to the monotonically decreasing pattern. The result of variance analysis showed that species richness (*p* < 0.01), abundance (*p* < 0.01) and Margalef abundance index (*p* < 0.05) at the <300 m altitude gradient presented significant differences compared to those at other altitude gradients. However, there were no significance differences between the Shannon–Wiener diversity index and the Pielou evenness index at the four altitude gradients.

### 3.3. Factors Associated with the Species Richness and Abundance of Birds

Through step regression of GLM models, we picked out the minimum environmental variables under each spatial scale (Table 3). The dispersion of the fitted model was tested by examining the residuals value (*p* > 0.05), and the models fit well.

To avoid the impact of spatial autocorrelation, we developed the spatial linear models (SLM) for the species richness and abundance with three lag distances on three spatial scales, respectively (Table 4). The result of the likelihood ratio test (LR test) shows that the model of the Margalef abundance index was not applicable (*p* < 0.001). The AIC values show that spatial scale of 750 m was better support the SLM models than other spatial scales. Meanwhile, the lag distance of 2 km was more supportive of the models than other lag distances. Then, we found that the SLM model with a spatial scale of 750 m and a lag distance of 2 km is the best model for both species and abundance, with the lowest AIC value. The Moran_I values show that there was no spatial autocorrelation in richness and abundance models. The z values of each environmental variables show that the season (*p* < 0.001), contagion index (*p* < 0.01), Shannon–Wiener landscape diversity index (*p* < 0.01) and average canopy height (*p* < 0.05) played important roles in explaining the variance of the species richness. However, season (*p* < 0.001), average canopy height (*p* < 0.001) and percentage of bamboo (*p* < 0.01) played important roles in explaining the variance of the species abundance. It is noteworthy that the altitude was present only in the SLM model of species richness. This shows that altitude has less influence on the species abundance in broad-leaved evergreen forests of Wuyishan National Park.

**Figure 3:**
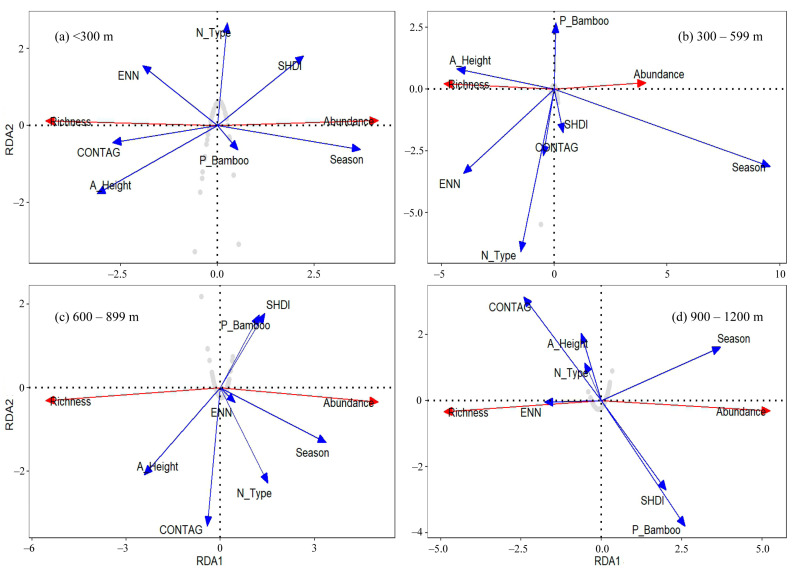
The result of redundancy analysis (RDA): all bird species richness and abundance with an explanatory variable during the four altitude gradients (<300 m, 300–599 m, 600–899 m, 900–1200 m). Gray point is the survey transect. SHDI is Shannon–Wiener landscape diversity index; A_Height is the average canopy height; P_Bamboo is percentage of bamboo; ED is edge density index; ENN is Euclidean nearest-neighbor distance index; CONTAG is contagion index; N_Type is the number of land cover types.

To analyze the effect of altitude, redundancy analysis (RDA) with seven explanatory variables (season, Euclidean nearest-neighbor distance index, contagion index, Shannon–Wiener landscape diversity index, percentage of bamboo, average canopy height and the number of land cover types) from the best-supported model for species richness and abundance was performed. We found that the influence of the environment variable was partly changed according to the altitude gradient (Table 5; Figure 3). The total explained variables created the highest contribution to changes in species composition at an altitude gradient of 900–1200 m, with a contribution of 36.92%. Despite this, in other altitude gradients, the contribution is much lower. The effect of season on species composition was significant (*p* < 0.05) in all four altitude gradients. Meanwhile, the effect of average canopy height (*p* < 0.05) was significant at an altitude gradient of 300–599 m and 600–899 m. These values demonstrated that species composition had an insignificant correlation with other landscape context variables.

## 4. Discussion

Differences in the habitat use and plasticity of birds can lead to differences in bird diversity across altitude gradients [46]. The species richness and abundance of birds in the evergreen broad-leaved forest of Wuyishan National Park conform to the monotonically decreasing pattern and were highest in the <300 m elevation region, forming a clear difference from other altitude gradients. Firstly, this is due to the higher habitat heterogeneity in the lower elevation areas of Wuyishan National Park, which provides more types of habitats for birds [61]. Secondly, it is due to the vertical migration of birds in autumn and winter, which causes alpine birds to come to lower elevations to overwinter [62,63]. Meanwhile, birds prefer to rely on surplus food generated by human activities because food resources are scarcer in winter [64].

Elevation, season and landscape interacted with each other to affect the bird community [9,45]. We also found that the species richness of birds was associated with altitude, season and landscape context because birds can flexibly use different types of the environment across seasons when resource availability and the annual stage of the bird life cycle change [65,66]. Obviously, the season is more significant than other explanatory variables. The current research on biodiversity patterns in landscape mainly focuses on the importance of habitat quantity and habitat configuration [67]. The amount of habitat is particularly important when habitat patches are isolated [68]. Our study confirmed that habitat configuration might be more important when the number of suitable habitats is large [69].

At the landscape level, we found that the average canopy height (A_Height) and contagion index (CONTAG) positively correlate with species richness and abundance. This is basically similar to the previous study. The tree height is closely related to vegetation succession and is an important indicator of forest age [70]. Previous studies have shown that forest age is positively correlated with bird diversity [71,72,73]. The contagion index (CONTAG) represents the convergence and connectivity of landscape. Generally, the high values indicate that patches in the landscape are clustered, forming good connectivity. On the contrary, low values indicates that the patches are scattered with high fragmentation. Due to connectivity, birds may access more habitat patches, increasing the amount of adjacent patches’ accessible habitat. Higher species richness and compositional shift resulted [74], and certain area-sensitive species can survive. However, one of the primary causes of the decline in bird populations is landscape fragmentation. The biodiversity of native forests may be greatly impacted by fragmentation, which may alter the species composition and richness, population abundance, species distribution and other factors [75,76]. In addition, regional differences may exist due to landscape features associated with environmental gradients. The composition of bird assemblages in different plots is usually similar [77]. Differences in landscape context can determine the relative abundance of each guild. Conversely, evidence suggests that guilds with wider habitat widths respond differently to landscape context [78].

Significant effects on the distribution of species and their richness are also caused by the composition (land-use percentage) and distribution of components [79]. Arbor forest landscapes are considered one of the most attractive for birds due to their complex vegetation structure, which can provide abundant food resources and ideal nesting conditions for birds [80]. The area of the arbor forests in the study area accounted for a high percentage, with mainly evergreen broad-leaved forests and a high number of bird species. However, an increase in bamboo was not conducive to an increase in the number of individual birds in the region, which may be due to the homogeneous structure of bamboo forests and the lack of food resources, mostly applied to avian bamboo specialists [81].

For even the most mature landscapes, landscape change is a continuous process. Our results rely on land cover data, and with the continuous updating of satellite remote sensing technology, high-resolution long-term monitoring of land cover through satellite remote sensing has also been achieved [25]. Therefore, it’s helpful to analyze the landscape context effect as the acquisition of land cover data will be faster and more effective than ever [23].

Wuyishan National Park has been protected since the establishment of the national nature reserve over 40 years ago, and it can be considered a representative of better secondary forest restoration. Therefore, in the forest restoration region, in addition to increasing the average canopy height and contagion index, reasonable measures should be taken in specific altitude gradients, such as controlling the area of bamboo forests and reducing fragmentation at altitude gradient of <300 m while increasing habitat heterogeneity at altitude gradients of >300 m.

## 5. Conclusions

In our study, we surveyed the bird communities at different altitude gradients in the subtropical evergreen broad-leaved forest of Wuyishan National Park. In summary, the result showed that (1) species of richness and abundance at <300 m altitude were highest among the four altitude gradients, and they showed more significant differences than those at other altitude gradients; (2) the species richness of birds was associated with altitude, season and landscape context, as the season was more significant than other explanatory variables; (3) at the landscape level, habitat configuration was more important, as the average canopy height and contagion index positively correlate with the species richness and abundance of birds at all four altitude gradients. In particular, the average canopy height presented significance at the 300–599 m and 600–899 m altitude gradients.

Therefore, accelerating the evolution of forest succession and improving the connectivity of patches while reducing the area of bamboo forests and fragmentation can effectively promote the recovery of bird diversity, especially in forest restoration regions.

## Figures and Tables

**Table 1:** Average values (±standard error) for variables of each altitude gradient (** is *p* < 0.01; * is *p* < 0.05).

Variables	Altitude Gradient	500 m			750 m			1000 m		
		Mean	SD	F Value	Mean	SD	F Value	Mean	SD	F Value
ED (m/ha)	<300 m	134.56	69.38	63.356 **	129.7	70.86	76.42 **	125.77	69.65	86.063 **
	300–599 m	58.44	31.81		52.08	30.06		48.64	28.28	
	600–899 m	61.43	37.44		54.55	30.4		49.81	26.53	
	900–1200 m	71.23	31.18		63.06	20.86		58.16	14.26	
LSI	<300 m	6.2	2.78	77.771 **	7.51	3.57	93.523 **	8.68	4.19	103.044 **
	300–599 m	3.16	0.94		3.49	1.14		3.86	1.35	
	600–899 m	3.4	1.33		3.78	1.43		4.13	1.51	
	900–1200 m	3.5	1.01		3.93	0.92		4.35	0.81	
ENN (m)	<300 m	83.35	80.68	2.956 *	68.54	49.79	11.11 **	64.56	34.45	33.232 **
	300–599 m	118.47	95.3		128.35	90.63		155.19	89.54	
	600–899 m	119.35	86.13		119.14	63.97		132.04	51.94	
	900–1200 m	134.82	170.73		112.92	56.52		105.9	29.3	
CONTAG (%)	<300 m	63.14	12.09	8.373 **	65.62	13.33	7.279 **	66.26	13.53	6.766 **
	300–599 m	71.59	14.17		73.09	13.86		74.09	14	
	600–899 m	65.35	19.06		69.96	14.24		71.68	13.36	
	900–1200 m	64.91	11.22		66.83	8.74		69.64	7.35	
SHDI	<300 m	0.82	0.33	8.766 **	0.81	0.34	8.341 **	0.81	0.34	8.18 **
	300–599 m	0.61	0.32		0.61	0.32		0.61	0.33	
	600–899 m	0.65	0.35		0.64	0.32		0.63	0.3	
	900–1200 m	0.73	0.24		0.71	0.18		0.67	0.13	
P_Arbor (%)	<300 m	59.17	26.04	14.58 **	61.68	24.43	13.11 **	63.17	23.02	12.065 **
	300–599 m	74.8	21.48		75.66	20.35		76.19	20.23	
	600–899 m	58.1	31.81		60.58	30.33		62.38	29.32	
	900–1200 m	62.7	18.54		67.76	13.37		71.79	10.66	
P_Shurb (%)	<300 m	17.53	15.43	23.402 **	17.2	12.96	36.439 **	17	11.53	52.176 **
	300–599 m	8.14	11.58		7.14	9.76		6.32	8.04	
	600–899 m	6.11	6		5.2	5.26		4.79	4.96	
	900–1200 m	4.26	3.23		3.29	1.95		2.79	1.35	
P_Bamboo (%)	<300 m	2.53	3.38	69.014 **	2.63	5.38	63.723 **	2.88	7.44	55.262 **
	300–599 m	10.79	14.84		11.31	14.08		11.93	14.17	
	600–899 m	33.96	27.53		32.63	26.64		31.53	25.91	
	900–1200 m	32.26	16.94		28.31	12.68		24.89	10.58	
A_Height (m)	<300 m	10.26	4.39	251.109 **	10.31	4.13	258.097 **	10.23	4.05	256.182 **
	300–599 m	19.07	4.69		19.53	4.5		19.97	4.38	
	600–899 m	25.14	3.22		24.96	3		24.63	2.79	
	900–1200 m	26.84	3.36		26.33	3.19		25.65	3.09	
N_Type	<300 m	4.41	1.3	11.671 **	4.76	1.22	20.103 **	4.88	1.29	12.964 **
	300–599 m	3.9	0.96		4.1	0.98		4.27	0.97	
	600–899 m	3.58	1.11		3.71	1.03		4.06	0.99	
	900–1200 m	3.56	0.61		3.69	0.47		3.94	0.56	

**Table 2:** The bird diversity index in different altitude gradient (capital letter is *p* < 0.01; lowercase letter is *p* < 0.05). H′ is Shannon–Wiener diversity index; J is Pielou evenness index; D′ is Margalef abundance index.

Altitude	Richness	Abundance	H′	J	D′
<300 m	20.54 ± 5.941 ^A^	153.01 ± 56.23 ^A^	1.298 ± 0.841	0.726 ± 0.613	1.136 ± 0.138 ^Aa^
300–599 m	18.4 ± 5.165 ^B^	134.81 ± 42.02 ^B^	1.397 ± 0.86	0.861 ± 0.753	0.997 ± 0.07 ^Ab^
600–899 m	17.2 ± 3.685 ^Bc^	126.35 ± 24.97 ^B^	1.455 ± 0.9	0.95 ± 0.789	0.758 ± 0.068 ^Bb^
900–1200 m	17.8 ± 4.529 ^Bc^	133.48 ± 33.85 ^B^	1.43 ± 0.982	0.962 ± 0.969	0.858 ± 0.107 ^Bb^

**Table 3:** The result of step regression with GLM models. SHDI is Shannon–Wiener landscape diversity index; A_Height is the average canopy height; P_Bamboo is percentage of bamboo; ED is edge density index; ENN is Euclidean nearest-neighbor distance index; CONTAG is contagion index; N_Type is the number of land cover types.

Spatial Scale	Response Variables	Environmental Variables	Dispersion Value	*p* Value
500 m	Richness	Season + SHDI + A_Height	0.99971	0.96
	Abundance	Season + P_Bamboo + A_Height	0.99291	0.904
	D	ED + SHDI + A_Height	0.99291	0.904
750 m	Richness	Altitude + Season + ENN + CONTAG + SHDI + P_Bamboo + A_Height + N_Type	0.98202	0.76
	Abundance	Season + ENN + CONTAG + P_Arbor + P_Bamboo + A_Height	0.98638	0.832
	D	ENN + CONTAG + ED + Altitude + SHDI	0.98856	0.856
1000 m	Richness	Altitude + Season + CONTAG + SHDI	0.99073	0.888
	Abundance	Season + ENN + P_Bamboo + A_Height	0.99073	0.888
	D	CONTAG + SHDI + Altitude	0.99291	0.904

**Table 4:** The z cores of SLM for species richness and abundance of birds in three spatial scales (500 m, 750 m and 1000 m). The lag distances are 1 km, 2 km and 3 km. (* Pr(>|z|) < 0.05, ** Pr(>|z|) < 0.01, *** Pr(>|z|) < 0.001). SHDI is Shannon–Wiener landscape diversity index; A_Height is the average canopy height; P_Bamboo is percentage of bamboo; ED is edge density index; ENN is Euclidean nearest-neighbor distance index; CONTAG is contagion index; N_Types is the number of land cover types. LR test is likelihood ratio test.

Variable	500 m			750 m			1000 m		
	1 km	2 km	3 km	1 km	2 km	3 km	1 km	2 km	3 km
Richness									
Altitude	-	-	-	−1.56	−1.381	−1.4	−3.220 **	−3.065 **	−3.042 **
Season	3.541 ***	3.505 ***	3.457 ***	3.557 ***	3.532 ***	3.494 ***	3.538 ***	3.487 ***	3.445 ***
ENN	-	-	-	−1.552	−1.437	−1.636	-	-	-
CONTAG	-	-	-	2.795 **	2.913 **	2.846 **	1.361	1.438	1.454
SHDI	2.504 *	2.307	2.137	2.720 **	2.981 **	2.991 **	3.506 ***	3.450 ***	3.545 ***
P_Bamboo	-	-	-	1.666	1.253	1.057	-	-	-
A_Height	−4.116 ***	−4.052 ***	−4.008 ***	−2.325 *	−2.315 *	−2.314 *	-	-	-
N_Type	-	-	-	−1.814	−1.895	−1.798	-	-	-
Moran_I	−0.003	0.002	0.001	−0.003	0.001	−0.001	−0.004	0.001	−0.001
AIC	2714.8	2713.5	2715.5	2714.6	2713.2	2714.1	2717.1	2717.7	2719.2
LR test value	12.154	13.471	11.39	8.4648	9.9031	8.9191	11.837	11.286	9.781
R^2^	0.157	0.159	0.154	0.173	0.176	0.173	0.156	0.154	0.149
Abundance									
Season	5.647 ***	5.660 ***	5.574 ***	5.649 ***	5.695 ***	5.647 ***	5.656 ***	5.683 ***	5.615 ***
ENN	-	-	-	−2.803 **	−2.563 *	−2.766 **	−1.436	−1.324	−1.421
CONTAG	-	-	-	1.716	1.454	1.653	-	-	-
P_Arbor	-	-	-	1.572	1.504	1.581	-	-	-
P_Bamboo	2.063 *	1.915	2.019 *	3.259 **	2.997 **	3.210 **	2.748 **	2.501 *	2.640 **
A_Height	−5.910 ***	−5.458 ***	−5.782 ***	−5.424 ***	−5.093 ***	−5.348 ***	−5.588 ***	−5.263 ***	−5.443 ***
Moran_I	−0.003	0.001	0	−0.003	0.001	0	−0.004	0.001	0
AIC	4653.3	4651.1	4653.8	4646	4645.1	4645.9	4649.2	4647.7	4649.1
LR test value	13.789	16.449	12.062	12.837	15.001	12.277	13.801	16.787	12.776
R^2^	0.136	0.142	0.135	0.16	0.163	0.16	0.147	0.152	0.148
Margalef abundance index (D′)									
Altitude	-	-	-	−2.808 **	−2.467 *	−2.545 *	−2.704 **	−2.431 *	−2.551 *
ENN	-	-	-	−1.250	−1.187	−1.607	-	-	-
CONTAG	-	-	-	1.877	2.264*	2.248 *	1.188	1.471	1.542
ED	−1.761	−1.370	−1.138	−1.718	−1.173	−0.846	-	-	-
SHDI	3.135 **	2.676 **	2.401 *	3.919 ***	3.848 ***	3.730 ***	3.184 **	3.263 **	3.370 ***
A_Height	−3.570 ***	−3.367 ***	−3.380 ***	-	-	-	-	-	-
Moran_I	−0.003	0.004	0.001	−0.002	0.004	−0.001	−0.003	0.004	−0.001
AIC	1210.3	1213	1214.7	1212	1214.2	1214.7	1212.3	1215.3	1216.3
LR test value	1.038 ***	3.247 ***	0.563 ***	0.018 ***	0.920 ***	0.104 ***	0.492 ***	2.320 ***	0.601 ***
R^2^	0.140	0.132	0.127	0.143	0.137	0.134	0.136	0.127	0.123

**Table 5:** Results of redundancy analysis (RDA). Explained variation (%): the proportion of variation that is explained by each measure. F: F statistics. P: *p*-value of F statistics. * *p* < 0.05; ** *p* < 0.01; *** *p* < 0.001. SHDI is Shannon–Wiener landscape diversity index; A_Height is the average canopy height; P_Bamboo is percentage of bamboo; ED is edge density index; ENN is Euclidean nearest-neighbor distance index; CONTAG is contagion index; N_Type is the number of land cover types.

Variables	<300 m			300–599 m			600–899 m			900–1200 m		
	Explained Variation (%)	F	*p*	Explained Variation (%)	F	*p*	Explained Variation (%)	F	*p*	Explained Variation (%)	F	*p*
Season	13.94%	4.79	0.029 *	15.95%	17.88	0.001 ***	13.09%	8.87	0.003 **	20.28%	6.96	0.01 **
ENN	3.84%	1.32	0.256	2.83%	3.18	0.065	0.26%	0.17	0.694	4.59%	1.58	0.2
CONTAG	3.82%	1.32	0.245	0.13%	0.15	0.763	0.16%	0.11	0.766	4.43%	1.52	0.197
SHDI	0.06%	0.02	0.929	0.12%	0.13	0.78	5.08%	3.44	0.062	0.40%	0.14	0.722
P_Bamboo	0.59%	0.20	0.648	0.08%	0.09	0.821	0.01%	0.01	0.957	3.00%	1.03	0.336
A_Height	3.31%	1.14	0.307	5.79%	6.48	0.003 **	7.08%	4.80	0.022 *	0.08%	0.03	0.878
N_Type	0.00%	0.00	0.996	0.22%	0.25	0.653	3.11%	2.10	0.149	4.15%	1.42	0.218
Total	25.56%	1.26	0.277	25.13%	4.02	0.001 ***	28.79%	2.79	0.007 **	36.92%	1.81	0.105

## Data Availability

The data used to support the findings of this study are available from the corresponding author upon request.
